# Proton pump inhibitors induce changes in the gut microbiome composition of systemic lupus erythematosus patients

**DOI:** 10.1186/s12866-022-02533-x

**Published:** 2022-04-27

**Authors:** Xian-Bao Li, Xiu-Jie Chu, Nv-Wei Cao, Hua Wang, Xin-Yu Fang, Yin-Guang Fan, Bao-Zhu Li, Dong-Qing Ye

**Affiliations:** 1 Department of Epidemiology and Biostatistics, School of Public Health, Anhui Medical University, Hefei, Anhui China; 2 Inflammation and Immune Mediated Diseases Laboratory of Anhui Province, Hefei, Anhui China

**Keywords:** Systemic lupus erythematosus, Gut microbiota, Proton pump inhibitors

## Abstract

**Background:**

Currently, few studies focus on the association between gut microbiota and systemic lupus erythematosus (SLE), and much less studies consider the effect of drug usage. Proton pump inhibitors (PPIs) are commonly used to treat drug-related gastrointestinal damage in SLE patients. Therefore, the purpose of this study is to examine the gut microbiota of SLE patients using PPIs.

**Methods:**

Fecal samples from 20 SLE patients with PPIs (P-SLE), 20 SLE patients without PPIs (NP-SLE) and 17 healthy controls (HCs) were obtained. The structure of the bacterial community in the fecal samples was analyzed by 16S rRNA gene sequencing. Redundancy analysis (RDA) was performed to observe the relationship between clinical variables and microbiome composition in P-SLE and NP-SLE patients. Based on the Kyoto Encyclopedia of Genes and Genomes (KEGG) database, functional capabilities of microbiota were estimated. Network analysis was performed to analyze the association of metabolic pathway alterations with altered gut microbiota in P-SLE and NP-SLE patients.

**Results:**

P-SLE patients exhibited increased alpha-diversity and an altered composition of the gut microbiota compared with NP-SLE patients. The alpha-diversity of NP-SLE patients was significantly lower than HCs but also of P-SLE patients, whose alpha-diversity had become similar to HCs. Compared with NP-SLE patients, the relative abundances of *Lactobacillus*, *Roseburia*, *Oxalobacter*, and *Desulfovibrio* were increased, while those of *Veillonella*, *Escherichia*, *Morganella*, *Pseudomonas* and *Stenotrophomonas* were decreased in P-SLE patients. RDA indicated that PPI use was the only significant exploratory variable for the microbiome composition when comparing SLE patients. KEGG analysis showed that 16 metabolic pathways were significantly different between NP-SLE and P-SLE patients. These metabolic pathways were mainly associated with changes in *Escherichia*, *Roseburia*, *Stenotrophomonas*, *Morganella* and *Alipipes* as determined by the network analysis.

**Conclusions:**

PPI use is associated with an improved microbiome composition of SLE patients as it 1) increases alpha-diversity levels back to normal, 2) increases the abundance of various (beneficial) commensals, and 3) decreases the abundance of certain opportunistic pathogenic genera such as *Escherichia*. Validation studies with higher patient numbers are however recommended to explore these patterns in more detail.

**Supplementary Information:**

The online version contains supplementary material available at 10.1186/s12866-022-02533-x.

## Background

Systemic lupus erythematosus (SLE) is a severe autoimmune disease that leads to the formation of immune complexes and inflammation in multiple organs in human body [1]. Although genetically induced risks have been proposed as the aetiology of SLE, environmental factors have also received great attention because of their contribution to the occurrence and development of SLE [2]. Gut microbiota as an internal environmental factor may be a missing link in SLE pathogenesis [3]. Studies have already shown significant changes in gut microbiota of SLE patients compared to healthy individuals [4, 5]. Nevertheless, perturbation of gut microbiota in SLE patients can be reversed by pharmacological treatment [6, 7]. Although studies have reported altered gut microbiota in SLE patients, few have assessed the association between gut microbiota and drug use in SLE treatment [8].

Various therapeutic agents have been used to achieve SLE remission, such as antimalarial agents, glucocorticoids, nonsteroidal anti-inflammatory drugs (NSAIDs), immunosuppressive agents, and B cell-targeting biological agents [8]. These drugs have been found to significantly alter structure of gut microbiota [7, 9, 10]. Glucocorticoids may reduce the expression of inflammatory cytokines by adjusting the structure of gut microbiota in SLE patients [7]. Therefore, we investigated effects of drugs on gut microbiota in SLE.

Proton pump inhibitors (PPIs) are generally used to treat gastric ulcers in SLE patients. PPIs are a class of acid-suppressant drugs that inhibit gastric acid secretion by covalently binding to hydrogen potassium ATPase [11]. Studies showed significant changes in abundance and diversity of gut microbiota in PPIs administered patients [12, 13]. The relative abundance of 20% bacterial taxa in patients administered with PPIs was significantly different from that in non-PPIs administered patients [13, 13]. Imhann et al. found a significant increase in the genera *Streptococcus*, *Enterococcus*, *Staphylococcus*, and *Escherichia coli,* among the patients treated with PPIs [14]. Thus, this study aims to evaluate the association between altered gut microbiota in SLE patients and PPIs use.

## Results

### Patients’ data and 16S rRNA gene sequencing

This study included 20 SLE patients with PPIs (P-SLE), 20 SLE patients without PPIs (NP-SLE) and 17 healthy controls (HCs). Table 1 showed basic information of three groups. Erythrocyte sedimentation rate (ESR), complement 3 (C3), complement 4 (C4), C-reactive protein (CRP), autoantibody status, and use of hydroxychloroquine, glucocorticoids, immunosuppressants, or NSAIDs did not significantly differ between P-SLE and NP-SLE patients. A total of 2,025,735 reads were obtained by filtering out ambiguous bases, homologous single bases and chimeras. Clean and optimized sequences of each sample were listed in Supplementary Table 1. A total of 345 operational taxonomic units (OTUs) were obtained. The shapes of species accumulation curves indicated that the most of relevant species detected at least once in all samples combined (Supplementary Fig. 1).

**Table 1:** Clinical characteristics of all subjects in this study

	HCs	SLE	
		P-SLE	NP-SLE
Fecal samples	17	20	20
Age, years, mean ± SD	30.12 ± 14.14	34.25 ± 10.54	35.95 ± 10.27
BMI, kg/m^2^, mean ± SD	22.48 ± 2.51	22.49 ± 3.39	23.16 ± 2.78
Disease duration, years, median (IQR)	-	1.5(0.15–4.7)	5.5(0.7–10.75)
SLEDAI, mean ± SD	-	9.65 ± 4.78	6.5 ± 4.51
ESR, mm/h, mean ± SD	-	48.8 ± 28.99	31.5 ± 20.68
CRP, mg/l, mean ± SD	-	12.08 ± 25.72	9.59 ± 20.63
C3, g/l, mean ± SD	-	0.64 ± 0.24	0.76 ± 0.34
C4, g/l, mean ± SD	-	0.10 ± 0.09	0.13 ± 0.08
Lupus nephritis, n (%)	-	9(45)	6(30)
Positive anti-dsDNA, n (%)	-	11(55)	14(70)
Positive anti-SSA, n (%)	-	14(70)	15(75)
Positive anti-SSB, n (%)	-	4(20)	6(30)
Positive anti-RNP, n (%)	-	11(55)	16(80)
Drugs use			
Hydroxychloroquine, n (%)	-	15(75)	18(90)
Glucocorticoid, n (%)	-	18(90)	17(85)
Immunosuppressant, n (%)	-	10(50)	6(30)
NSAIDs, n (%)	-	3(15)	6(30)

*HCs* healthy controls, *PPIs* Proton pump inhibitors, *P-SLE* SLE patients who received PPIs, *NP-SLE* SLE patients who didn’t receive PPIs, *IQR* interquartile range, *DAI* Disease activity index, *NSAIDs* Non-Steroidal Anti-inflammatory Drugs

### Changes of microbial diversity in SLE patients with/without PPIs administration

**Fig. 1:**
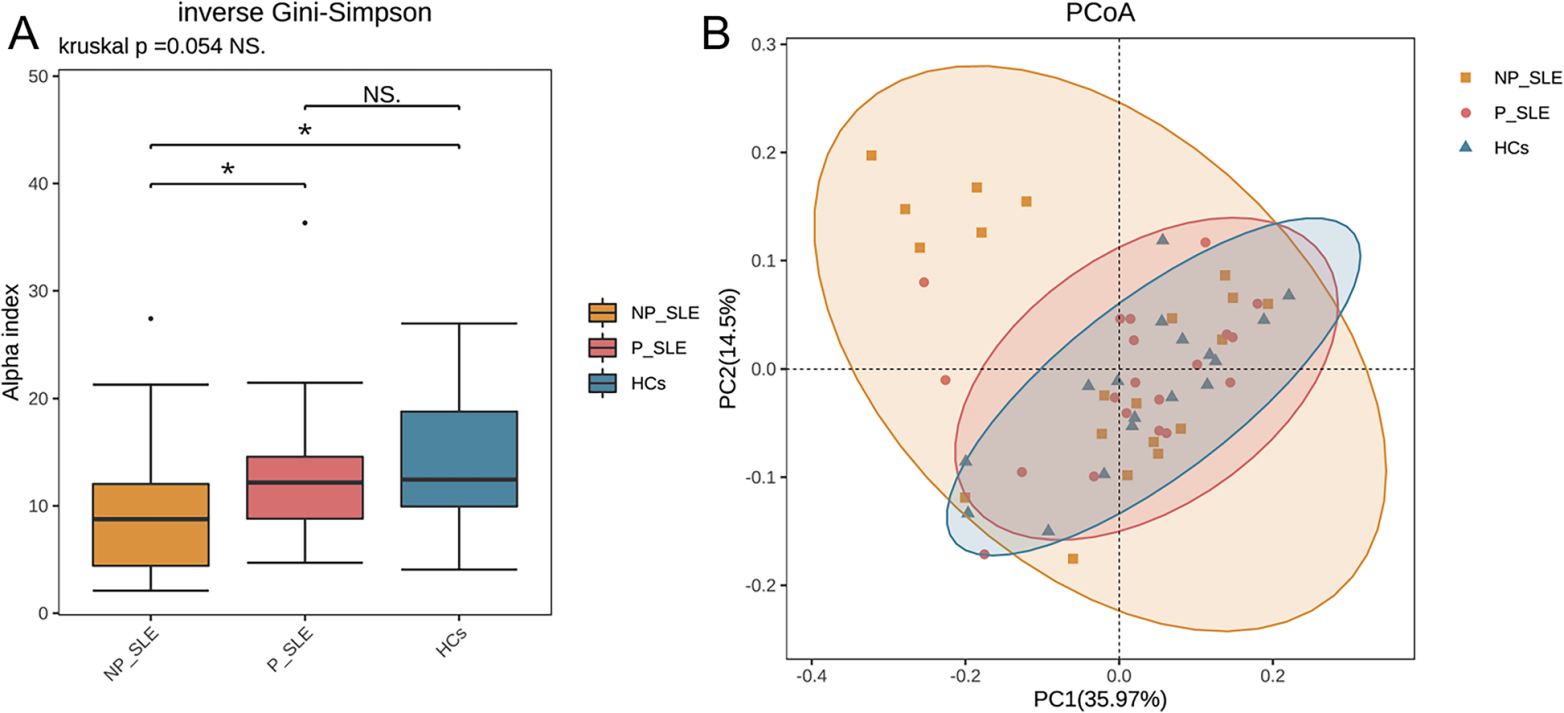
Diversities of the gut microbiota among P-SLE patients, NP-SLE patients and HCs. **A** inverse Gini-Simpson index; The middle line in the box plot represents the median value, and the box is drawn from the 25% to75% quartiles. **B** Principal coordinate analysis (PCoA) of the bacterial community structures in HCs, P-SLE patients and NP-SLE patients. HCs: healthy controls; P-SLE: SLE patients with PPIs; NP-SLE: SLE patients without PPIs

The alpha-diversity of gut microbiota in NP-SLE patients was decreased compared with HCs. However, P-SLE patients had a higher alpha-diversity compared with NP-SLE patients. The inverse Gini-Simpson indices were significantly lower in NP-SLE patients (Fig. 1A). The alpha-diversities of NP-SLE patients were lower than those of HCs (Fig. 1A, Supplementary Fig. 2). However, the alpha diversity indices of P-SLE patients were higher than those of NP-SLE patients (Fig. 1A, Supplementary Fig. 2). In addition, there was no difference in alpha diversity between P-SLE patients and HCs.

Beta-diversity analysis, a measure for the distance in the microbial composition between groups of samples or within a group of samples. It showed that the variation within NP-SLE patients was higher than those within other groups, and indicated that NP-SLE patients were more different from P-SLE patients and HCs than the different between P-SLE patients and HCs. In this study, principal coordinate analysis (PCoA)1 and PCoA2 accounted for 35.97% and 14.5% of the variation, respectively (Fig. 1B). The PCoA analysis results showed that NP-SLE patients tended to separate from HCs and P-SLE patients, and the distribution of NP-SLE patients and HC samples was similar. Therefore, a permutational multivariate analysis of variance (PERMANOVA) analysis was performed, and the results showed that the difference between any two groups was not statistically significant (*P* > 0.05) (Supplementary Table 2). We calculated the Bray–Curtis distance between every sample pair and in this way simply calculate the distance between groups shown in a box-plot (Supplementary Fig. 3).

### The abundance of microbiota was altered in SLE patients compared with HCs

**Fig. 2:**
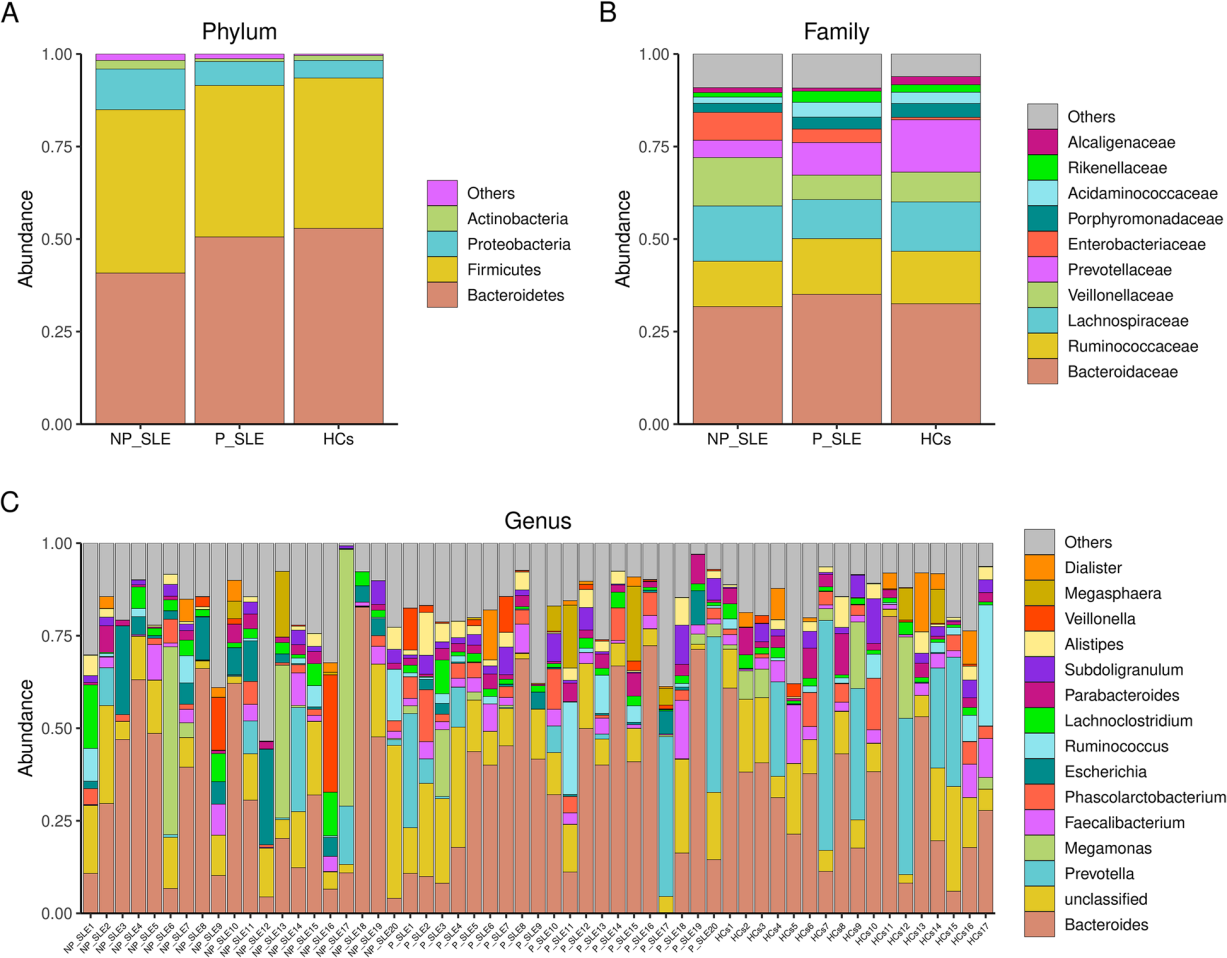
Characteristics of the microbial composition in SLE patients with PPI use. **A** Relative abundance of the dominant bacteria at phylum level in the gut microbiota of SLE patients with or without PPIs use and the HCs group; **B** Relative abundance of the dominant bacteria at phylum level in the gut microbiota of SLE patients with or without PPIs use and the HCs group; **C** Relative abundance of the dominant bacteria at genus level in the gut microbiota of SLE patients with or without PPIs use and the HCs group. HCs: healthy controls; P-SLE: SLE patients with PPIs; NP-SLE: SLE patients without PPIs

We examined the taxonomic composition and relative abundance of gut microbiota in P-SLE patients, NP-SLE patients, and HCs at different taxonomic levels. A total of 10 phyla were identified in SLE patients and HCs, among which the three most abundant phyla were Bacteroides, Firmicutes, and Proteobacteria (Fig. 2A). At the phylum level, no statistical differences were observed between three groups (data not shown). The five dominant, most abundant families in the fecal microbiota were *Bacteroidaceae*, *Ruminococcaceae*, *Lachnospiraceae, Veillonellaceae* and *Prevotellaceae* (Fig. 2B). As shown in Fig. 2B, the relative abundances of *Veillonellaceae* and *Enterobacteriaceae* in NP-SLE patients were higher than those in P-SLE patients and HCs, while the abundance of *Prevotellaceae* was lower than those in P-SLE patients and HCs. In addition, the gut microbiota compositions of major genera among different groups were shown in Fig. 2C.

The relative abundances of microbiome in NP-SLE patients and HCs were different. At the genus level, the relative abundance of *Rothia*, *Morganella*, *Escherichia*, *Pseudomonas*, *Stenotrophomonas*, *Veillonella*, and *Enterococcus* was higher in NP-SLE patients than in HCs (Supplementary Table 3). Conversely, *Roseburia*, *Oxalobacter*, *Desulfovibrio*, and *Dialister* were more abundant in HCs than in NP-SLE patients (Supplementary Table 3).

Abundances of various genera were also different between P-SLE and NP-SLE patients. *Desulfovibrio*, *Oxalobacter*, *Roseburia*, *Streptococcus* and *Lactobacillus* were higher in P-SLE patients, whereas the relative abundance of *Veillonella*, *Escherichia*, *Pseudomonas*, *Stenotrophomonas*, and *Morganella* was lower, mimicking the difference between NP-SLE patients and HCs (Supplementary Table 4). Compared with HCs, P-SLE patients differed slightly in various different genera. *Streptococcus* and *Rothia* were enriched in P-SLE patients, while *Fusicatenibacter* and *Parasutterella* were enriched in HCs (Supplementary Table 5). Differences on other phylogenetic levels were detailed in Supplementary Tables 3, 4 and 5. The relative abundance of all bacterial groups for all 57 samples can be obtained from Supplementary Table 6.

**Fig. 3:**
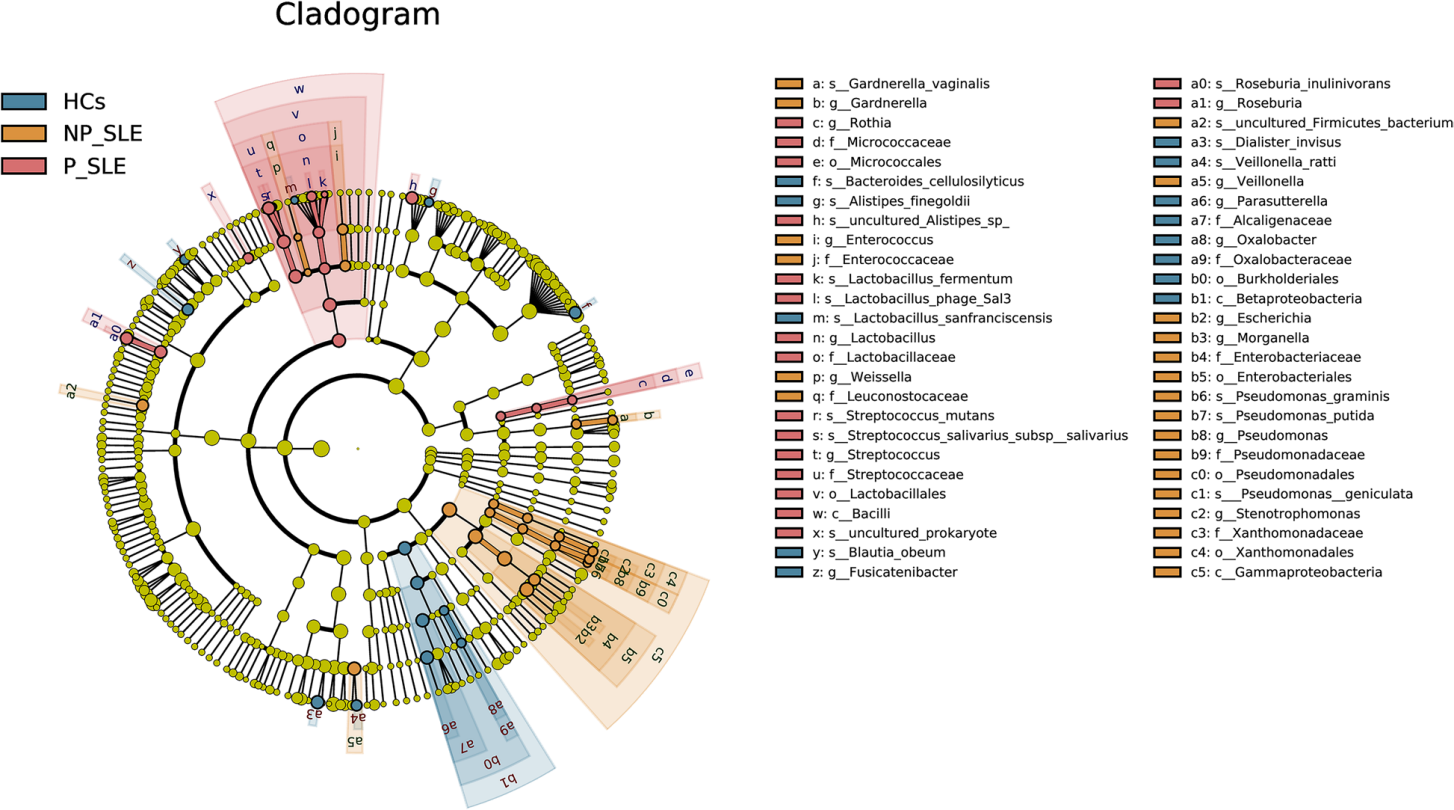
Compositions of the gut microbiota among SLE patients and HCs. LEfSe analysis was performed to identify differentially abundant taxa by the phylogenetic tree; Linear discriminant analysis (LDA) results were showed by LDA score. HCs: healthy controls; P-SLE: SLE patients with PPIs; NP-SLE: SLE patients without PPIs

To further clarify the bacterial taxa, we used linear discriminant analysis effect size (LEfSe) to compare composition and relative abundance of gut microbiota between SLE patients and HCs for different taxonomic categories (Fig. 3). Tendencies for increased abundance of *Alcaligenaceae*, *Oxalobacteraceae*, *Fusicatenibacter*, and *Oxalobacter* were observed in HCs compared to NP-SLE and P-SLE patients. The abundance of *Streptococcaceae*, *Lactobacillaceae*, *Micrococcaceae*, *Roseburia*, and *Rothia* were higher in P-SLE patients than in NP-SLE patients and HCs. The abundance of *Enterobacteriaceae*, *Enterococcaceae*, *Leuconostocaeae*, *Pseudomonadaceae*, *Xanthomonadaceae*, *Veillonella*, *Stenotrophomonas*, and *Morganella* were enriched in NP-SLE patients compared with P-SLE patients and HCs.

### Clinical variables associated with microbial changes in P-SLE and NP-SLE patients

**Fig. 4:**
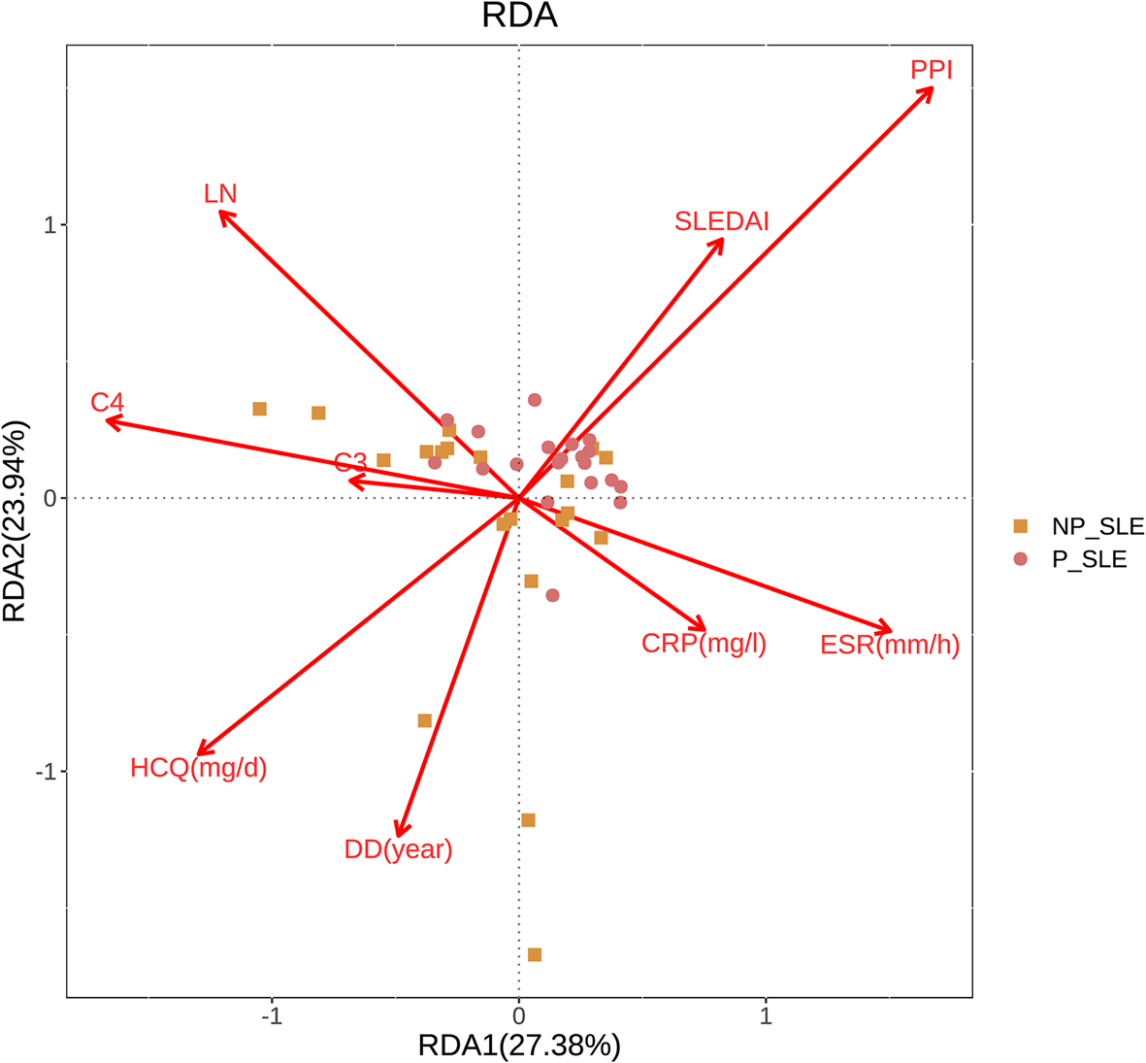
Redundancy analysis based on Bray–Curtis dissimilarity. RDA analysis indicated that only PPIs were significant explanatory variables for microbiome composition (*P* < 0.05). CRP: C-reactive protein; ESR: Erythrocyte sedimentation rate; HCQ: Hydroxychloroquine; SLEDAI: Systemic Lupus Erythematosus Disease Activity Index 2000; DD: Disease duration; LN: Lupus nephritis; C3: Complement 3; C4: Complement 4; P-SLE: SLE patients with PPIs; NP-SLE: SLE patients without PPIs

Additional analyses were conducted to ascertain whether the difference in gut microbiota composition between P-SLE and NP-SLE patients were also significantly affected by clinical factors other than PPIs use. As age and BMI were matched, they were excluded in the redundancy analysis (RDA). RDA indicated that PPIs use was the only significant explanatory variable for microbiome composition (*P* < 0.05). hydroxychloroquine, the SLE activity index (SLEDAI), lupus nephritis, disease duration, C4, ESR, CRP, and C3 did not significantly influence the microbiome composition (Fig. 4).

### Altered pathways and their relationships with microbiota in NP-SLE and P-SLE patients

Based on the Kyoto Encyclopedia of Genes and Genomes (KEGG) database, phylogenetic investigation of communities by reconstruction of unobserved states (PICRUSt) analysis showed that 16 KEGG pathways were statistically different in P-SLE and NP-SLE patients by LEfSe analysis (*P* < 0.05) (Supplementary Fig. 4). Among the 16 altered pathways, carbon fixation pathways in prokaryotes, cell growth and death, thiamine metabolism, immune system, DNA replication, drug metabolism other enzymes, NOD like receptor signalling pathway and plant pathogen interaction pathways were increased in P-SLE patients. The nitrotoluene degradation, biofilm formation by Escherichia coli, propanoate metabolism, pentose phosphate pathway, phosphotransferase system PTS, ABC transporters, membrane transport and environmental information processing pathways were reduced in P-SLE patients.

**Fig. 5:**
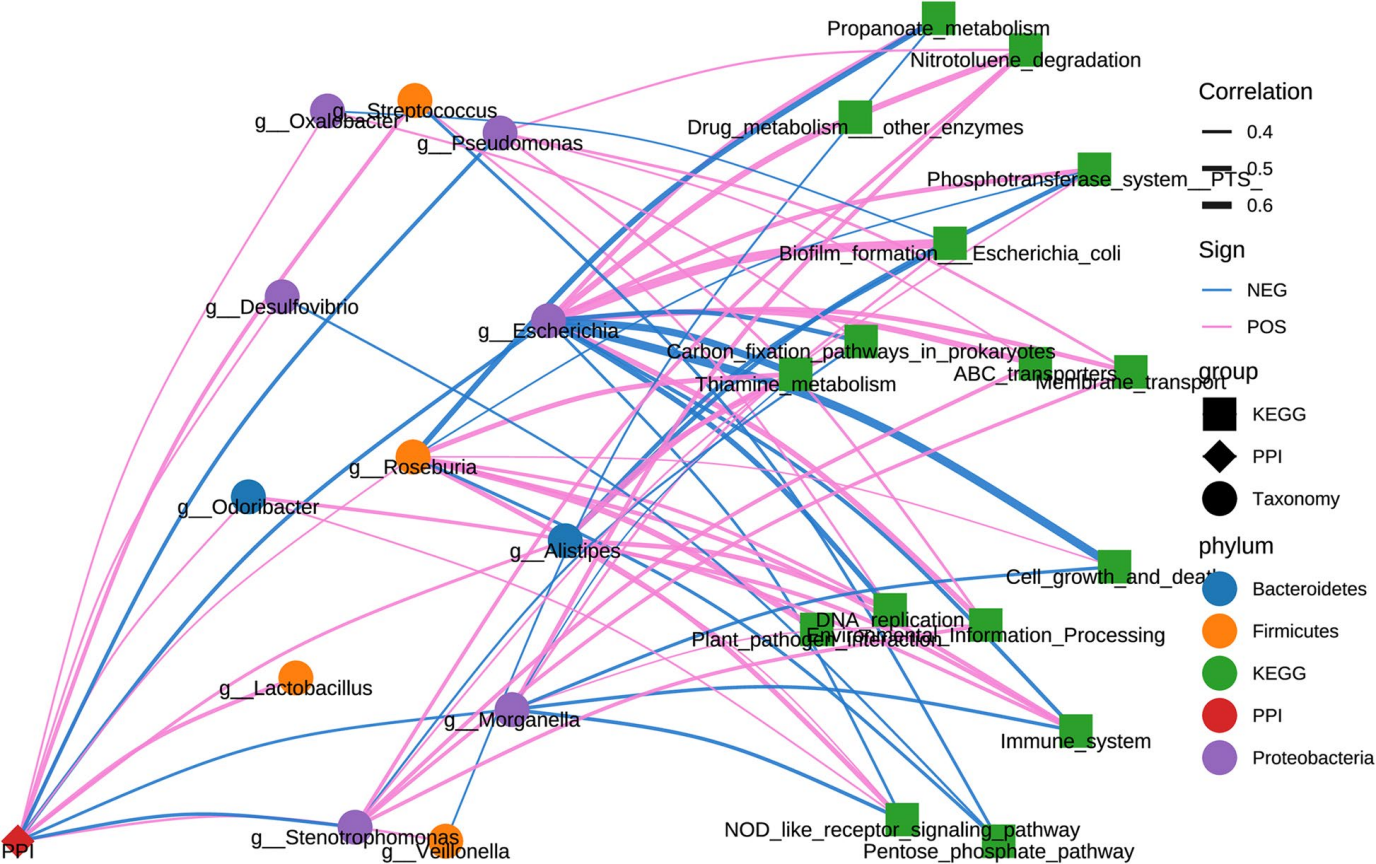
Association network analysis of PPIs, microbiota and KEGG pathways in P-SLE and NP-SLE patients. The diamonds represent PPI, the circles represent species, the squares represent KEGG category, and the different colors of species represent different phyla-level classifications; the thickness of the lines represents the strength of correlation, pink represents positive correlation, and blue represents negative correlation

To further explore the association altered metabolic pathways with microbiome, a network analysis was conducted. Figure 5 shows that PPIs affect metabolic pathways through *Escherichia*, *Roseburia*, *Stenotrophomonas*, *Morganella* and *Alipipes* in SLE patients.

## Discussion

First, our study demonstrated that both diversity and composition of microbiota were different between P-SLE and NP-SLE patients, whereas the characteristics of P-SLE patients were similar to those of HCs. Second, changes in gut microbiota of P-SLE and NP-SLE patients were mainly caused by PPI use. Third, PPI use was associated with altered microbial metabolic pathways in SLE patients. Overall, in SLE patients the microbiota composition was more variable, and frequently lacked a cohesive structure dominated by commensal bacteria. PPI use tended to improve their situation to a certain extent in that their gut microbiota composition became comparable again with HCs.

Differences in the relative abundance of gut microbiota between P-SLE and NP-SLE patients were observed in our study. Based on the sequencing results, NP-SLE patients had low alpha diversity compared to P-SLE patients and HCs (*P* < 0.05). Another study also found that PPI use could increase the alpha diversity of gut microbiota [15]. The oftentimes dysbiotic-like gut microbiota composition in SLE patients, reflected by increased numbers of *Enterobacteriaceae* and/or small intestinal and oral bacteria, is in turn a reflection of the disturbance of normal trophic networks of commensal (beneficial) microorganisms. Higher alpha-diversity values are typically representative of such complex networks of commensal bacteria. Increased alpha diversity after PPI use, represented also by the increase of particular commensal groups, therefore appears favourable.

Although no statistical differences in beta-diversity were found in our study, beta-diversity of NP-SLE patients were quite dissimilar to those of both P-SLE patients and HCs. A common cause of such a high dissimilarity was a high abundance of the *Enterobacteriacea*e family. A high *Enterobacteriaceae* abundance typically is indicative of the presence of opportunistic pathogenic genera such as *Escherichia* [16]. SLE is a severe disease with immune complexes and inflammation in multiple organs. Except PPIs, various unknown factors affect the state of SLE and gut microbiota in SLE patients. In this study, it failed to find other clinical factors due to smaller effect sizes than PPIs use, which could be further analysed with a larger effect size in future studies. Thus, the probability of finding SLE patients with similar states is small. The Anna Karenina principle tends to apply to these samples, where healthy gut microbiome compositions tend to be more alike whilst unhealthy gut microbiome compositions are often out of order in their own unique way.

Most previous studies have confirmed that gut microbiota were altered in SLE patients [17–20]. Our results showed that the relative abundances of *Veillonella*, *Escherichia*, *Morganella*, *Pseudomonas*, and *Stenotrophomonas* were higher in NP-SLE patients. Similarly, Li et al. found that the relative abundance of *Veillonella* increased in the gut microbiota of SLE patients, whereas the relative abundance of *Roseburia* decreased [17]. In a previous study, significant depletion of *Lactobacillus* was found in lupus-susceptible female mice compared to that in age-matched healthy female mice [6]. In addition, studies have shown that *Escherichia* infection is common in patients with SLE [18–20].

PPI use is associated with altered composition of gut microbiota [21–23]. Studies have shown differences in different phylogenetic levels after the use of PPIs. The abundance of Bacilli, Lactobacillales, *Lactobacillaceae*, *Streptococcaceae*, *Lactobacillus*, and *Lactobacillus salivarius* which were reported decreasing in SLE patients were relatively normal in P-SLE patients as compared with HCs. Furthermore, the relative abundance of *Veillonella* and *Escherichia* was reduced in patients receiving PPIs.

In all, it appears that PPI use in SLE seem to be associated with a “healthier” microbiome composition. However, it is only an association and not causality. Since we do not know exactly what “healthy” is in terms of microbiome composition, more well-designed perspective studies are needed to study the causal relationship between PPI use and microbiome composition changes in SLE patients.

Furthermore, the use of PPIs in SLE patients was associated with alterations in 16 metabolic pathways. The changes of metabolic pathways in NP-SLE and P-SLE patients were mainly related to *Escherichia*, *Roseburia*, *Stenotrophomonas*, *Morganella* and *Alipipes*. These taxa may be prominent predictors of SLE health. It certainly indicates PPIs might play a role in altered gut microbiota in SLE patients. Further studies using metagenomics, metatranscriptomics, and metabolomics methods are needed to explore the impact of PPIs on the functional capacity of the gut microbiome.

Notably, previous studies have found that the relative abundance of *Prevotellaceae* were higher in SLE patients than in HCs [5, 18]. In contrast, in our results, *Prevotellaceae* were reduced in NP-SLE patients, restored to some extent in P-SLE patients, and were highly abundant in HCs. However, a small sample size causes the problem of “enterotype” associated differences, such as the presence/absence of members of *Prevotella* (and their close relatives) is more difficult to capture with just a limited number of samples. In addition, PPIs have been used as short-term drugs to improve the gastrointestinal symptoms of SLE. However, some studies have found that the long-term use of PPIs could have adverse effects [24, 25]. Future research might hence benefit from longer trials where PPI treated SLE patients are followed for longer periods of time collecting samples at various points in time.

This study also found Archaea in human digestive tract. Archaea are prokaryotes widely studied in environmental microbiology, but play an important role in human health. *Methanobrevibacter smithii* is the methanogens usually detected in human gut. Evidence indicated a close connection between *Methanobrevibacter smithii* and dysbiosis of the digestive tract [26–28]. But the abundance of Archaea is different in population. One reason for the difference may be existence of the competing group-sulfate-reducing bacteria (SRB). *Methanobrevibacter smithii* and SRB are the two groups competing for hydrogen in human body [29, 30]. This study didn’t measure the presence of SRB and culture *Methanobrevibacter smithii*. In this study, *Methanobrevibacter smithii* were detected in eight samples. Future studies using shotgun sequencing or culture should be conducted to explore the role of *Methanobrevibacter smithii* in SLE.

## Conclusions

PPIs use is associated with an improved microbiome composition of SLE patients as it 1) increases alpha-diversity levels back to normal, 2) increases the abundance of various (beneficial) commensals and 3) decreases the abundance of certain opportunistic pathogenic genera such as Escherichia. Validation studies with higher patient numbers are however recommended to explore these patterns in more detail.

## Methods

### Patients and healthy controls

SLE patients were recruited from the First Affiliated Hospital of the Anhui Medical University. They were diagnosed by chief physicians of the department of rheumatology and immunology, and met the American College of Rheumatology revised classification diagnostic criteria or the Systemic Lupus International Collaborating Clinics criteria. HCs were recruited from communities. All participants ensured that they did not receive antibiotics, probiotics, or synbiotics for at least three months. However, patients with other autoimmune diseases, chronic metabolic diseases, chronic infectious diseases, other dermatoses, or cancers were excluded from this study.

All participants were female. NP-SLE patients were defined as SLE patients who did not use PPIs before enrolment in the study, while P-SLE patients were defined as SLE patients who used PPIs (such as rabeprazole, 20 mg/day). NP-SLE patients, P-SLE patients, and HCs were matched for age and BMI. All fecal samples were collected before 8 am, immediately transferred to the laboratory, and stored at -80 °C. All participants provided informed consents.

### Extraction and determination of bacteria

DNA of fecal samples from subjects was extracted by QIAamp DNA Stool Mini Kit. To prepare gut microbiome library for sequencing, 16S rRNA were amplified at V3 to V4 hypervariable region by polymerase chain reaction (PCR). According to Illumina high-throughput sequencing requirements, two-way sequencing was performed, and primers were designed using (357f-806R) as the forward primer (5'-ACTCCTACGGRAGGCAGCAG-3’) and reverse primer (5'-GGACTACHVGGGTWTCTAAT-3') [31]. The two-step PCR amplification method was used to build a library. The first-step PCR amplification was performed employing a step cycling protocol consisting of 94 °C for 2 min, 25 cycles of 94 °C for 30 s, 56 °C for 30 s, and 72 °C for 30 s, ending with the final elongation at 72 °C for 5 min and 10 °C heat preservation. All PCR products were recovered using the AxyPrepDNA gel recovery kit, and quantified using FTC-3000TM real-time PCR instrument (Shanghai Fengling Biological Technology Co.,Ltd. China). Then secondary PCR amplification was subjected, and the adapters were added which were required for sequencing. The second-step PCR amplification was performed employing a step cycling protocol consisting of 94 °C for 2 min, 8 cycles of 94 °C for 30 s, 56 °C for 30 s, and 72 °C for 30 s, ending with the final elongation at 72 °C for 5 min and 10 °C heat preservation. Then, PCR products were recycled using AxyPrepDNA gel recovery kit (AXYGEN, U.S.A.).

### Sequence analysis

For sequencing of 16S rRNA gene-based amplicons, the amplicon library was prepared using a Novaseq 6000 SP 500 Cycle Reagent Kit (Illumina, USA). Low-quality, ambiguous reads or homologous, including mismatching reads and raw reads shorter than 50 bp, were firstly filtered for the following assembly. The maximum allowable error ratio of an overlap region was 0.2. According to overlap relationship between the paired-end reads, pairs of reads were merged into a sequence and the minimum overlap length was 10 bp. Trimmomatic (Version 0.35) was used to control sequence quality [32]. Paired-end clean reads were further merged using FLASH (Version 1.2.11) [33].

OTUs clustering on the clean tags was performed, and then OTU species classification was completed by annotating OTU. USEARCH (Version V8.1.1756) was used to cluster the assembled sequences into OTU. The representative sequence of OTU was obtained by UPARSE software clustering under 97% similarity. The chimera generated by PCR amplification was removed from the OTU representative sequence by UCHIME software (http://drive5.com/uparse/). The USEARCH_GLOBAL method was used to align all sequences back to the OTU representative sequences, and the statistical table of the abundance of each sample in each OTU was obtained. Next, species annotation was performed by comparing the OTU representative sequence with the database (Silva128) [34] through mothur (Version 1.33.3) software, and the confidence threshold was set to 0.6.

### Statistical analysis

The species richness and evenness of microbial communities reflected by alpha-diversity. The alpha-diversity of the gut microbiota between groups was compared using Chao1, inverse Gini-Simpson index and Shannon index. Microbial differences between samples were analyzed by beta-diversity. The differences between microbial communities were determined by PCoA using weighted UniFrac dissimilarity distance metric. The alpha-diversity and beta-diversity were analyzed using mothur software. The Wilcoxon rank sum test was applied to determine the significance of microbial diversity between groups and samples. PERMANOVA was used to compare differences between groups [35]. LEfSe analysis was used to identify significant enrichment of gut microbiota in different groups [36]. Linear discriminant analysis (LDA) was used to estimate the effect size of each distinctively abundant taxon. Nonparametric factorial Kruskal–Wallis (KW) sum-rank test was used to detect the taxa with significant abundance differences in R 3.6.1. Taxa with *P* value < 0.05 (KW test) and LDA score (log10) greater than 2.0 were considered as significantly enriched taxa.

Furthermore, based on KEGG database and KEGG orthologs, PICRUSt analysis was conducted to predict possible metabolic pathways of gut microbial in P-SLE and NP-SLE patients [37]. Based on Spearman associations, a network analysis was conducted to explore correlations between PPIs and microbiota (genus level) and KEGG pathways by R 3.6.1. And graph version 2.0.3 was performed for visualization. The figure only showed taxa with spearman correlation coefficient greater than 0.3 and *P* < 0.05.

In addition to the effect of PPIs on the gut microbiota, clinical indicators may also affect structure and abundance of gut microbiota, such as SLEDAI, the course of the disease, ESR, etc. Therefore, RDA analysis based on Bray Curtis dissimilarity was performed to observe relationships between clinical variables and microbiome composition in SLE patients [38].

Mean and standard deviation were used to descript continuous variables. Frequency and percentage were used to descript classifies variables. These were conducted by SPSS 23.0.

## Supplementary Information

**Additional file 1: Figure 1.** The species accumulation curves of this sequencing study.

**Additional file 2: Figure 2.** The alpha-diversity of the gut microbiota among P-SLE patients, NP-SLE patients and HCs.

**Additional file 3: Figure 3.** The Bray-Curtis distance.

**Additional file 4: Figure 4.** Featured microbial functions between P-SLE patients, NP-SLE patients and HCs group.

**Additional file 5.**

## Data Availability

Data are available in a public, open access repository. You can get data from ScienceDB (https://www.scidb.cn/s/nyEJ3q; DOI: https://www.doi.org/10.11922/sciencedb.01459).

## Abbreviations

SLE: Systemic lupus erythematosus
PPIs: Proton pump inhibitors
HCs: Healthy controls
P-SLE: SLE patients with PPIs
NP-SLE: SLE patients without PPIs
PCR: Polymerase chain reaction
OTUs: Operational taxonomic units
LEfSe: Linear discriminant analysis effect size
KW: Kruskal–Wallis
LDA: Linear Discriminant Analysis
KEGG: Kyoto Encyclopedia of Genes and Genomes
Kos: KEGG orthologs
PICRUSt: Phylogenetic investigation of communities by reconstruction of unobserved states
PCoA: Principal coordinates analysis
PC1: Principle component 1
PC2: Principle component 2
PERMANOVA: Permutational multivariate analysis of variance
RDA: Redundancy analysis
CRP: C-reactive protein
ESR: Erythrocyte sedimentation rate
