# Supplementary figures and images for: Proton pump inhibitors induce changes in the gut microbiome composition of systemic lupus erythematosus patients
Source: BMC Microbiol. 2022 Apr 27;22:117. doi: 10.1186/s12866-022-02533-x (PMC9043501; doi:10.1186/s12866-022-02533-x)

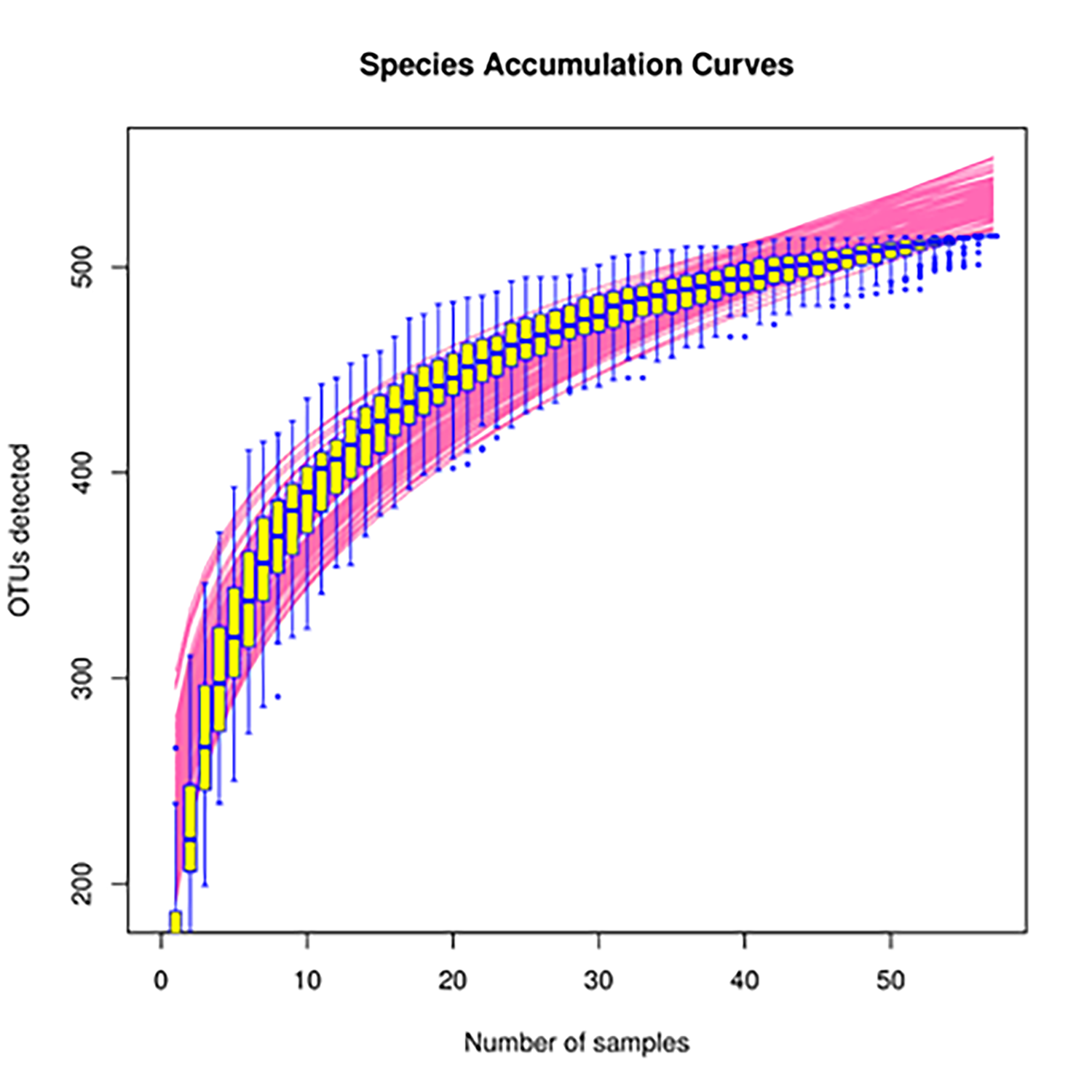

Supplement: Supplementary file 1 — Additional file 1: Figure 1. The species accumulation curves of this sequencing study. [file 12866_2022_2533_MOESM1_ESM.tiff]
